# Multiple congenital ocular anomalies in Icelandic horses

**DOI:** 10.1186/1746-6148-7-21

**Published:** 2011-05-26

**Authors:** Lisa S Andersson, Jeanette Axelsson, Richard R Dubielzig, Gabriella Lindgren, Björn Ekesten

**Affiliations:** 1 Department of Animal Breeding and Genetics, Swedish University of Agricultural Sciences, Box 597, SE-751 24 Uppsala, Sweden; 2 Department of Pathobiological Sciences, School of Veterinary Medicine, Comparative Ocular Pathology Laboratory of Wisconsin, University of Wisconsin-Madison, 2015 Linden Drive, Madison, WI 53706-1102, USA; 3 Department of Clinical Sciences, Swedish University of Agricultural Sciences, Box 7054, SE-750 07 Uppsala, Sweden

## Abstract

**Background:**

Multiple congenital ocular anomalies (MCOA) syndrome is a hereditary congenital eye defect that was first described in Silver colored Rocky Mountain horses. The mutation causing this disease is located within a defined chromosomal interval, which also contains the gene and mutation that is associated with the Silver coat color (*PMEL17*, exon 11). Horses that are homozygous for the disease-causing allele have multiple defects (MCOA-phenotype), whilst the heterozygous horses predominantly have cysts of the iris, ciliary body or retina (Cyst-phenotype). It has been argued that these ocular defects are caused by a recent mutation that is restricted to horses that are related to the Rocky Mountain Horse breed. For that reason we have examined another horse breed, the Icelandic horse, which is historically quite divergent from Rocky Mountain horses.

**Results:**

We examined 24 Icelandic horses and established that the MCOA syndrome is present in this breed. Four of these horses were categorised as having the MCOA-phenotype and were genotyped as being homozygous for the *PMEL17 *mutation. The most common clinical signs included megaloglobus, iris stromal hypoplasia, abnormal pectinate ligaments, iridociliary cysts occasionally extending into the peripheral retina and cataracts. The cysts and pectinate ligament abnormalities were observed in the temporal quadrant of the eyes. Fourteen horses were heterozygous for the *PMEL17 *mutation and were characterized as having the Cyst-phenotype with cysts and occasionally curvilinear streaks in the peripheral retina. Three additional horses were genotyped as *PMEL17 *heterozygotes, but in these horses we were unable to detect cysts or other forms of anomalies.

One eye of a severely vision-impaired 18 month-old stallion, homozygous for the *PMEL17 *mutation was examined by light microscopy. Redundant duplication of non-pigmented ciliary body epithelium, sometimes forming cysts bulging into the posterior chamber and localized areas of atrophy in the peripheral retina were seen.

**Conclusions:**

The MCOA syndrome is segregating with the *PMEL17 *mutation in the Icelandic Horse population. This needs to be taken into consideration in breeding decisions and highlights the fact that MCOA syndrome is present in a breed that are more ancient and not closely related to the Rocky Mountain Horse breed.

## Background

**Figure 1:**
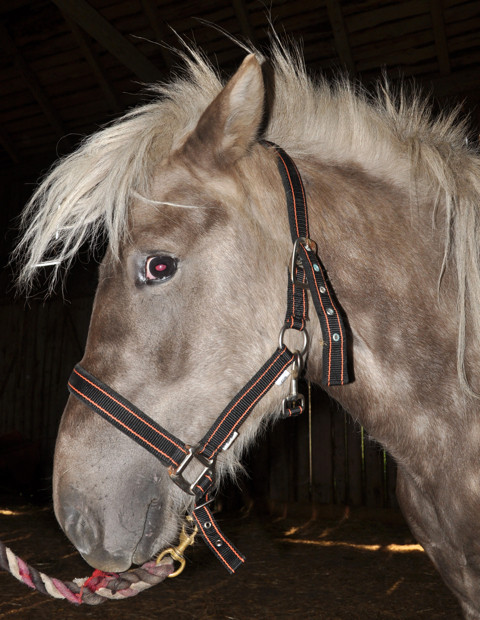
**An Icelandic horse with the Silver coat colour**. This horse is genetically black but the hairs have been lightened as a result of the *PMEL17 *mutation. This horse is homozygous for the mutation and displayed the MCOA-phenotype. Note that the pupil is poorly dilated even though a mydriatic has been applied repeatedly one hour before the photo was taken.

Multiple congenital ocular anomalies (MCOA) syndrome is a hereditary condition consisting of a wide range of ocular defects. It was first described in the Rocky Mountain Horse breed [1]. Most affected horses have a coat color called Silver (Figure 1). This color is associated with a missense mutation in *PMEL17 *and has a dominant mode of inheritance [2]. The mutation causes a dilution of eumelanin (black and brown pigment), most noticeable in mane and tail, into gray or white. The body of the horse becomes slightly diluted in color and often displays a dappled pattern. The *PMEL17 *mutation does not have an obvious effect on phenomelanin (red and yellow pigment), making it virtually impossible to detect it phenotypically in horses lacking dark pigment, for example chestnuts, white grays, palominos, pearl- and champagne-colored horses. These horses must be genotyped in order to determine if they are carriers of the mutation. The *PMEL17, exon 11*, mutation has been detected in an ancient sample recovered from South Siberia. Radiocarbon dating of this sample revealed that the Silver phenotype was present as early as 800 years BC [3]. It is still unclear if the *MCOA *locus and *Silver *locus are two separate but closely linked loci, or if only one mutation is present with pleiotropic effects, influencing both coat color dilution and ocular development [4].

Horses with the MCOA syndrome can be categorized into two separate groups depending on the severity of disease [5]. Horses that are heterozygous for the disease-causing allele have less severe ocular anomalies, mainly displaying peripheral cysts of the iris, cillary body or retina (Cyst-phenotype). Horses that are homozygous for the mutation have multiple anomalies, including fluid-filled cysts of the iris, ciliary body or retina, retinal dysplasia, megaloglobus, miotic pupils, iris stromal hypoplasia and cataract (MCOA-phenotype) [1,6]. Hence, the mutation displays a dose effect, were double mutants have more serious defects than horses carrying only one copy of the disease-causing allele [7].

Extensive breeding of horses with the desirable Silver coat color has lead to a high frequency of MCOA syndrome in Rocky Mountain horses. It has been argued that the ocular defects are caused by a recent mutation that is restricted to horses that are related to the Rocky Mountain Horse breed. Isolated findings of MCOA syndrome have been reported in the related Kentucky Mountain Saddle Horse, Mountain Pleasure Horse and Morgan Horse breeds [1,8]. Two MCOA cases have also been reported in the American Shetland pony [9], however this breed was developed by crossing imported Shetland ponies to finer-built domestic breeds [10].

In this study, we describe identical lesions in Icelandic horses, a breed developed in relative isolation on Iceland during the last 1000 years [11].

## Results

**Figure 2:**
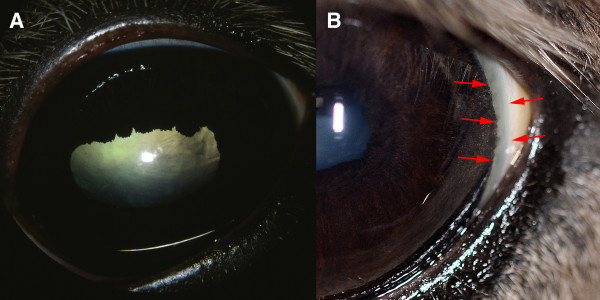
**Iridal abnormalities and cataracts in the MCOA-phenotype**. A) Iris stromal hypoplasia with miotic pupils with poor or no response to tropicamide was seen in all horses with the MCOA-phenotype. Cortical and nuclear cataractous changes in the lens contributed to the visual impairment. The horse in figure B) shows a regional abnormality of the pectinate ligament heralded by a widened, white perilimbal zone temporally (red arrows).

**Figure 3:**
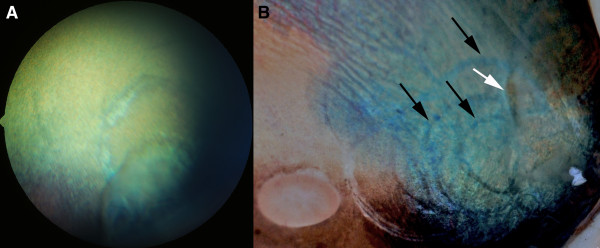
**Cystic lesions extending into the retina**. A) Cysts involving the peripheral retina in the temporal quadrant or curvilinear demarcation lines and focal areas of retinal atrophy were occasionally seen in both *PMEL17 *mutation heterozygotes and homozygotes. B) Post-mortem specimen showing multiple demarcation lines (black arrows) extending towards the optic nerve head and an area where the retina is still detached (white arrow).

We have examined 24 Icelandic horses and established that the MCOA syndrome is present in this breed. Four of the horses were categorized as having the MCOA-phenotype, which is the most severe form of the MCOA syndrome. This was in agreement with their genotype as they were homozygous for the exon 11, *PMEL17 *mutation (Figure 1). An abnormal corneal contour, megaloglobus was observed bilaterally in two horses with the MCOA-phenotype. Iridal stromal hypoplasia was found in all four horses. Miotic pupils that were irresponsive to light stimuli and repeated administration of tropicamide were seen in two horses, whereas the other two horses in this group had slightly miotic pupils that constricted when light was shone into the eye and instillation of tropicamide caused some, but not normal dilation of the pupils (Figure 2a). A widened, densely white perilimbal zone indicating iridocorneal angle anomalies was evident in the temporal quadrant in two of these horses (Figure 2b). Nuclear cataracts extending into the epinuclear and cortical areas were present in three homozygote MCOA horses examined, but not in a 12 month-old mare. All four horses with the MCOA-phenotype had multiple translucent, fluid-filled iridociliary cysts in the temporal quadrant of the eye. Involvement of the peripheral retina, always in the temporal quadrant, was seen as ophthalmoscopically visible cysts extending over the ora serrata or demarcation lines and focal retinal atrophy where the neuroretina previously had been detached (Figure 3).

Light-microscopic examination of one globe showed redundant duplication of non-pigmented ciliary body epithelium, sometimes forming cysts bulging into the posterior chamber. In the peripheral retina localized areas of retinal atrophy and loss of photoreceptors were seen.

**Figure 4:**
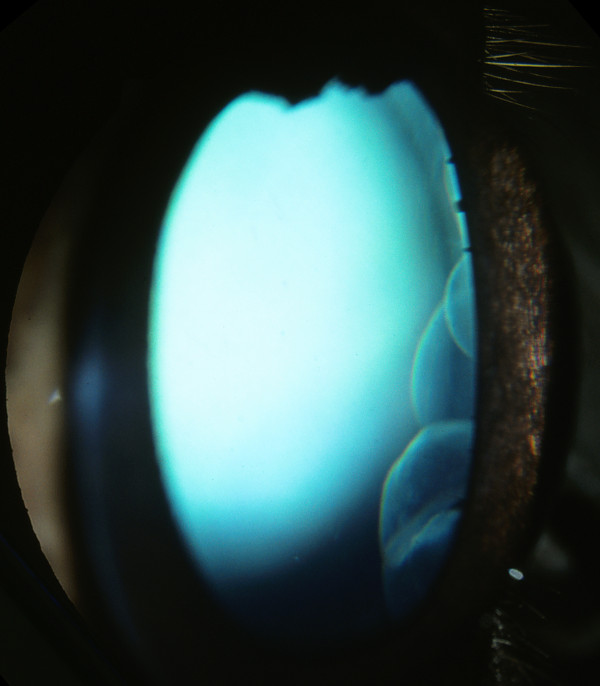
**Multiple iridociliary cysts in an Icelandic horse with the exon 11, *PMEL17 *mutation**. Multiple iridociliary cysts in the temporal quadrant were seen in all homozygotes and most heterozygotes. The size of the cysts ranged from a few millimetres in diameter to well over a centimetre.

Fourteen horses were classified with the Cyst-phenotype, and were shown to be heterozygous for the *PMEL17 *mutation. All of these horses showed translucent, fluid filled cysts, ranging from single small cysts in both eyes of one individual, to large lobulated cysts (Figure 4) with or without curvilinear streaks extending into the temporal retina. All horses with the Cyst-phenotype showed normal pupillary light reflexes, dazzle reflexes and menace responses and normal dilation of the pupil after tropicamide application, contrary to the MCOA-phenotype horses.

Three additional horses displayed the Silver phenotype, were heterozygous for the *PMEL17 *mutation, but did not display any detectable clinical signs of the Cyst-phenotype. Three non-Silver Icelandic horses were examined as controls. None of these displayed the Cyst- or the MCOA-phenotype.

Numerous pigmented folds indicating multifocal retinal dysplasia in the dorsal tapetal fundus were frequently observed in horses with the Cyst- or the MCOA-phenotype.

## Discussion

In this study we have shown that the MCOA syndrome is segregating with the *PMEL17 *mutation in the Icelandic Horse population. This makes the hypothesis that the *MCOA *mutation has recently arisen unlikely. Icelandic horses are believed to be descendant from horses brought to Iceland by the Vikings around 900 AD. They were possibly interbred later with ponies brought over from Ireland/ United Kingdom, which were the ancestors of the Shetland, Highland, and Connemara ponies. The Icelandic parliament banned import of horses into Iceland in 982 AD, and consequently the breed has been isolated for more than 1000 years.

It is probably not a coincidence that MCOA syndrome was first found in the Rocky Mountain Horse breed. Stallions and mares of this breed expressing the Silver phenotype have been used extensively in breeding. As the frequency of the Silver allele increased in the population, so did the frequency of the double mutants (homozygotes). In homozygous individuals, the ocular defects become the most obvious. This is reflected both in a phenotypic characteristic with bulging eyes (megaloglobus) and in an increased number of horses with impaired vision. In other breeds of horses where the mutation is not as common, carriers are most likely to be heterozygous [12]. This poses a particular problem to breeders as ocular anomalies in heterozygotes are usually only detected after careful examination by a trained ophthalmologist. Therefore, breeding societies may be unaware of the prevalence of the disease in their Silver horses, which warrants further clinical investigations.

We could not detect any clinical signs of MCOA syndrome in three *PMEL17 *heterozygous horses. This has also been reported in previous studies [4,5]. To date, we do not know if this was caused by limitations in our detection method or if it was due to non- penetrance of the mutation. However, horses without detectable cysts, that carry the *PMEL17 *mutation, still produce affected offspring [5].

Horses with the MCOA-phenotype are at particular risk of having impaired vision, and difficulties in adapting to changing light conditions are probably a common phenomenon in these horses. Some individuals have more severe impairment of their vision, causing abnormal behavior and an inability to perform. *MCOA *and *PMEL17 *are tightly linked, so breeding *PMEL17 *mutation carriers only to known non-carriers would practically eliminate the risk of producing horses with vision threatening abnormalities caused by this syndrome.

## Conclusion

MCOA syndrome is segregating with the *PMEL17 *mutation in the Icelandic Horse population. This should be taken in to consideration for breeding decisions and highlights the fact that the MCOA syndrome is present in a breed that is not closely related to the Rocky Mountain breed of horses.

## Methods

We have examined a total of 24 Icelandic horses. Horses were certified by the Swedish breed registry and included 19 females and 5 males (Table 1). Twenty-one horses had a Silver coat colour and three were non-Silver. The Silver colour could be traced back to five different stallions. The age of horses ranged from 1-23 years old (median: 5 years).

**Table 1:** Age and gender of Icelandic horses grouped according to their *PMEL17 *genotype

Genotype	Median age (and range) [years]	Mares / stallion / geldings
*PMEL17 +/+*	7 (1-15)	2/2/0

*PMEL17 +/-*	6 (2-20)	14/0/3

*PMEL17 -/-*	17 (14-23)	3/0/0

Plus denotes a mutant allele, whilst minus denotes wild-type.

Blood samples were collected at the time of clinical examination and DNA was extracted using a GeneMole robot (Mole Genetics, Lysaker, Norway). All horses were genotyped for the mutation in *PMEL17*, exon 11, that is associated with the Silver coat color with pyrosequencing as described in Brunberg et al [2].

One 18 month-old stallion with the MCOA-phenotype and severe visual impairment was euthanized according the owners request. The left eye was enucleated, hemisectioned and fixed in 4% glutaraldehyde. The globe was then submitted for light-microscopic examination.

Pupillary light reflexes, dazzle reflexes and menace responses were examined in all horses. The anterior and posterior segments of the eyes were examined by slit-lamp biomicroscopy (Kowa SL-14, Kowa Co. Ltd., Tokyo, Japan) and indirect ophthalmoscopy (Heine 500, Heine Optotechnik, Herrsching, Germany) before and after induction of mydriasis with tropicamide eye drops (Mydriacyl 0.5%, Alcon Sverige AB, Stockholm, Sweden). The examinations were approved by the Ethics Committee for Animal Experiments in Uppsala, Sweden and informed consent was received from all horse owners.

## Authors' contributions

LSA: planned the study, genotyped the horses, took part in the fieldwork and drafted the manuscript. JFA: took part in the fieldwork and drafted the manuscript. RD: performed the light microscopy. GL: planned the study and revised the manuscript. BE: Planned the study, performed all ophthalmologic examinations and revised the manuscript. All authors have read and approved the final manuscript.
